# Nasal nitric oxide testing for allergic rhinitis patients: Systematic review and meta‐analysis

**DOI:** 10.1002/iid3.439

**Published:** 2021-05-05

**Authors:** Bingbing Wang, Zhenchao Wu, Feifei Wang, Zuojuan Yin, Lei Shi, Yi Liu

**Affiliations:** ^1^ Department of Pulmonary and Critical Care Medicine Shandong Provincial Hospital Affiliated to Shandong University Jinan Shandong China; ^2^ Cheeloo College of Medicine Shandong University Jinan Shandong China; ^3^ Department of Pulmonary and Critical Care Medicine Shandong Provincial Hospital Affiliated to Shandong First Medical University Jinan China; ^4^ Shandong Key Laboratory of Infectious Respiratory Disease Jinan Shandong China; ^5^ Department of Pulmonary and Critical Care Medicine The Third Affiliated Hospital of Shandong First Medical University (Affiliated Hospital of Shandong Academy of Medical Sciences) Jinan Shandong China; ^6^ Department of Otolaryngology—Head and Neck Surgery Shandong Provincial Hospital Affiliated to Shandong University Jinan Shandong China; ^7^ Department of Otolaryngology—Head and Neck Surgery Shandong Provincial Hospital Affiliated to Shandong First Medical University Jinan Shandong China

**Keywords:** allergic rhinitis, meta‐analysis, nasal nitric oxide

## Abstract

**Background:**

Nasal nitric oxide (nNO) levels in allergic rhinitis (AR), healthy people or nonallergic rhinitis (NAR) have shown contradicting results in previous studies. By meta‐analysis, we reviewed studies that measured nNO in AR patients to assess nNO's ability to discriminate AR from healthy people or NAR.

**Methods:**

We systematically searched PubMed, Cochrane, Embase, Ovid, Web of Science, Wanfang Data, CNKI until December 15, 2020. Differences were expressed as standardized mean differences (SMD) with 95% confidence interval (CI), by random‐effects method.

**Results:**

A total of 10 original studies with 561 AR patients, 327 healthy controls, 123 NAR patients were included in the narrative synthesis and 9 studies in the meta‐analysis. nNO in AR was significantly increased compared with healthy controls (SMD: 0.989; 95% CI: 0.402, 1.576; *p* = .001) or NAR (SMD: 0.680; 95% CI: 0.101, 1.259; *p* = 0.021). However, subgroup analysis based on measuring process and patient characteristics showed that no significant differences were detected in nNO between AR patients with nasal polyps or sinusitis or marked ostial obstruction and healthy controls.

**Conclusions:**

nNO is a potential indicator for recognizing AR. Nasal polyps, sinusitis and marked ostial obstruction should be considered before nNO is applied to detect AR.

## Graphical abstract

The meta analysis shows that nasal nitric oxide (nNO) in allergic rhinitis (AR) is significantly higher than healthy people and nonallergic rhinitis. However sinusitis, nasal polysis and marked ostial obstruction should be taken into consideration before nNO is applied to detect AR.

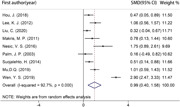

## 1 INTRODUCTION

Allergic rhinitis (AR) is a disease characterized by sneezing, itching, nasal congestion, and rhinorrhea following exposure of allergens. AR will detriment patients' efficiency of work and study, decline their quality of life and impact on asthma control level in AR patients combined with asthma, causing heavy healthcare economic burden.^1^ Allergic Rhinitis and its Impact on Asthma (ARIA) states that golden standard in AR diagnosis include demonstration of skin‐prick testing for allergens or the serum immunoglobulin E (IgE) tests.^2^

Nitric oxide (NO) is a free radical gas, playing an important role in many biological mechanisms. In respiratory system, NO is continuously released from upper and lower airway and soaringly released following proinflammatory cytokines and stimuli inducement. Fractional exhaled nitric oxide (FeNO) has been used as a noninvasive tool to reflect eosinophilic inflammation in lower airway diseases. For example, high level of FeNO suggests possibility of asthma.^3^ Moreover, FeNO is also a good indicator to monitor glucocorticoid treatment.^4^, ^5^, ^6^ Similarly, several studies indicated that nasal nitric oxide (nNO) could be used to predict AR.^7^, ^8^, ^9^ Contradictorily, some studies suggested that nNO in AR was not significantly different from healthy people.^10^, ^11^ Therefore, we undertook a systematic review and meta‐analysis on the nNO's ability to discriminate AR from healthy controls or nonallergic rhinitis (NAR).

## 2 METHODS

### 2.1 Data sources and searches

Our methods have been described detailly in the published protocol (PROSPERO registration: CRD42020160578). We systematically searched following databases until December 15, 2020: PubMed, Cochrane, Embase, Ovid, Web of Science, Wanfang Data, CNKI in accordance with the Preferred Reporting Items for Systematic Review and Meta‐Analyses for Diagnostic Test Accuracy. The search strategy used the following terms: “allergic rhinitis” AND “nasal nitric oxide” found within all fields. There was no constraint on the publication language or study design during searching.

### 2.2 Study selection

Studies were included if they measured nNO in AR patients and healthy controls with information about nNO analyzer, sampling technique, sampling rate and AR diagnostic criteria. Studies were excluded if any of the following were presented (1) number of AR patients was less than 10; (2) the procedure of nNO measurement did not follow American Thoracic Society (ATS)/ERS Recommendations for Standardized Procedures for the Online and Offline Measurement of Exhaled Lower Respiratory Nitric Oxide and Nasal Nitric Oxide, 2005;^12^ (3) AR diagnosis did not meet criteria described in ARIA guidelines.^2^, ^12^

### 2.3 Selection process

After duplicate article exclusion, B.W. and Z.W., two of the authors, independently analyzed the found articles and carried out data extraction. Information like clinical characteristics of subjects, NO analyzer, NO sampling rate, sampling technique, AR diagnosis, nNO value and so forth was collected. If disagreement came up, a third investigator (Y.L.) was consulted, decision would be finally made by consensus.

### 2.4 Quality assessment

The Quality Assessment of Diagnostic Accuracy Studies‐2 (QUADAS‐2) tool was used to evaluate the methodological quality of each study.^13^ The tool was explicitly developed to estimate the quality of diagnostic test from four domains (patient selection, index test, reference standard and flow/timing). Each domain was graded as low, high, or unclear risk.

### 2.5 Data synthesis and analysis

Studies reporting nNO values with mean and *SD* were included in the meta‐analysis. Otherwise, they would be excluded from meta‐analysis but still in qualitative synthesis. The reported nNO concentration (ppb) was converted into nl/min by formula ppb × sampling rate (L/min) to keep consistent between studies using different sampling rates.^14^ Data were analyzed using STATA 16.0. Differences between AR and healthy controls or NAR were expressed as standardized mean differences (SMD) with 95% confidence interval (CI). Random‐effects models were used to calculate summary effects across the studies. We also assessed studies for heterogeneity by *χ* ^2^ Cochran's *Q* test and *I* ^2^ statics. In detail, *I* ^2^ = 0% indicates no heterogeneity; 25%, low; 25%–50%, moderate; and more than 50%, high heterogeneity.^15^ Sensitivity analysis was performed by eliminating studies with high risk of bias. Subgroup analysis was applied in terms of patient characteristics and index test characteristics. Publication bias was assessed by funnel plot (SMD on the x‐axis against 1/*SE* of the SMD on the y‐axis), Egger test and the Begg and Mazumdar test. A *p* < .10 is considered statistically significant.^16^, ^17^

## 3 RESULTS

### 3.1 Study selection

**Figure 1:**
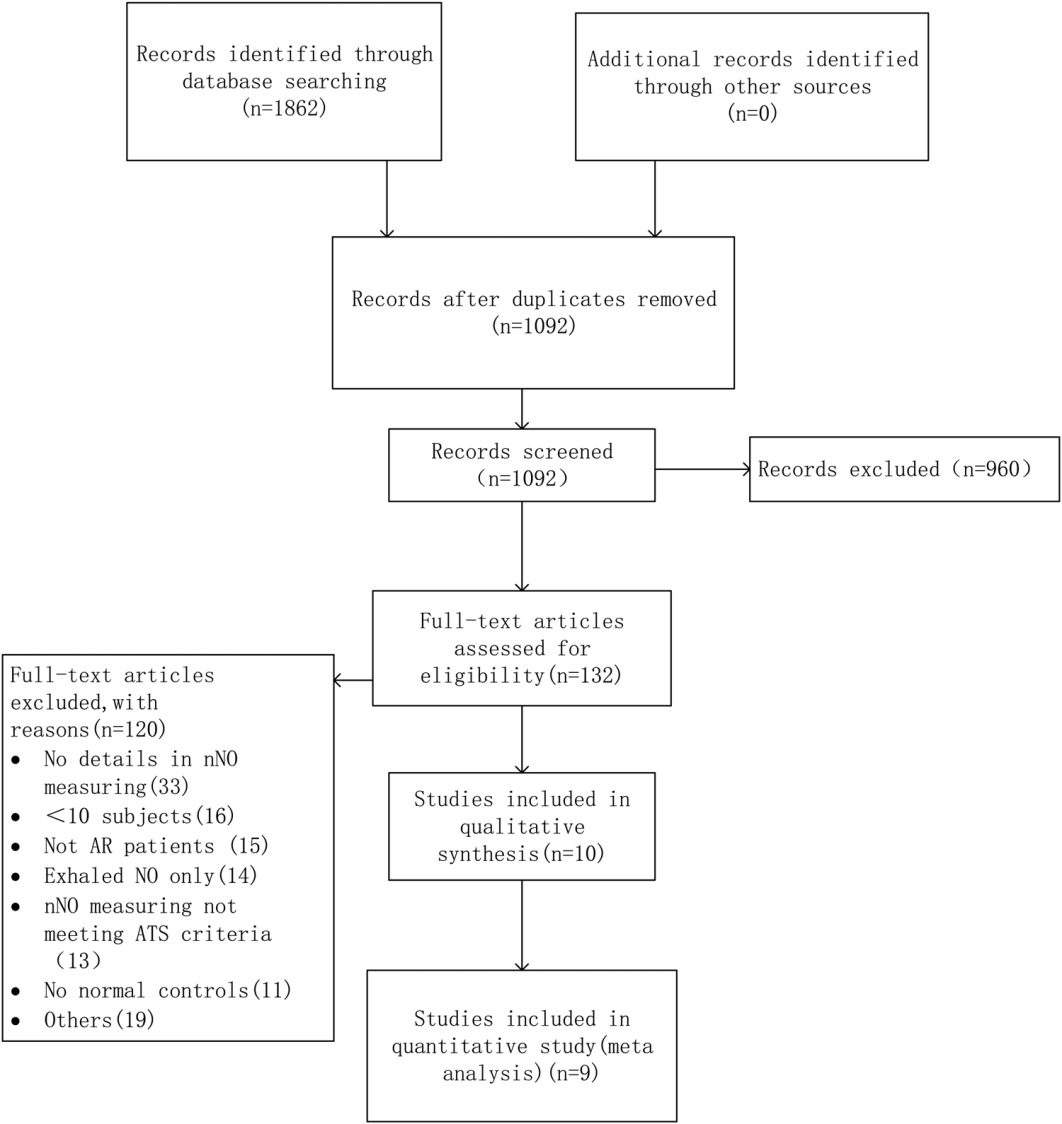
Summary of evidence search and selection

In total, 1862 records were identified through a generalized search of all publications related to AR and nNO. After removing duplicates, 1092 records were screened by title and abstract. After screening titles and abstracts, 132 potentially eligible studies were selected for full review. Finally, 10 original research studies^7^, ^8^, ^11^, ^18^, ^19^, ^20^, ^21^, ^22^, ^23^, ^24^ were included in the narrative synthesis and 9 studies^7^, ^8^, ^18^, ^19^, ^20^, ^21^, ^22^, ^23^, ^24^ in the meta‐analysis (Figure 1).

### 3.2 Study characteristics

The main demographic, study, index tests characteristics were presented in Tables 1 and 2. All studies included were case‐control studies. A total of 1011 participants were enrolled, 561 AR patients, 327 normal controls, 123 NAR patients. The age ranged from 4 to 50 years old. A total of 35.4%–73.1% was male gender. AR diagnosis was consistent with the guideline of ARIA,^2^ including seasonal AR and perennial allergic rhinitis (PAR) types. The diagnostic criteria were a typical history of allergic symptoms and diagnostic tests including skin prick tests or the blood specific IgE. Although some patients were not mentioned the status of AR,^7^, ^8^, ^20^, ^22^, ^23^ the rest AR patients were clear stated symptomatic or not. The prevalence of asthma was 42.7%–100% in two studies,^20^, ^22^ while asthma patients were excluded in the rest studies. Most studies prohibited AR medication before measuring nNO, which included corticosteroids, antihistamines, etc.^7^, ^8^, ^18^, ^19^, ^20^, ^21^, ^22^, ^23^, ^24^

**Table 1:** Patient and study characteristics

Study, year	Location	Patients, total	AR status	Ages	Gender, n male (%)		Asthma (%)	Medication
Henriksen (1999)^11^	Norway	AR 46 (Seasonal19, Seasonal + perennial27), HC 12	Symptomatic	AR: 16.4 (13–20); HC: 17.8 (16–19)	29/58 (50.0%)		0	Antihistamines
Palm (2003)^18^	Sweden	AR 18, HC 18	Symptomatic	AR: 32 (21–50); HC: 30 (20–46)	18/36 (50%)		0	No steroid, no antihistamines
Makris (2011)^19^	Greece	SAR 26, HC 15	Symptomatic	AR 28.4 (16–47); HC 37.1 (27–56);	22/41 (53.7%)		0	No corticosteroids, no antihistamines
Lee (2012)^7^	Korea	AR 35, patients with deviated nasal septa as HC 34	Not mentioned	AR: 22.7 ± 8.7; HC: 26.9 ± 11.0	42/69 (60.9%)		0	No nasal medication
Suojalehto (2014)^20^	Finland	AR 89, NAR 44, HC 42	Not mentioned	AR: 32.9 ± 1.3; NAR: 33.2 ± 1.5; HC: 33.5 ± 1.8	62/175 (35.4%)		42.7	No nasal steroids
Nesic (2016)^8^	Serbia	AR 23, HC 10	Not mentioned	AR: 33.4 ± 11.1; HC: 33.3 ± 8.4	18/33 (55%)		0	No steroids
Hou (2018)^21^	China	AR 75, HC 31	Symptomatic	AR: 36.24 ± 10.96; HC: 35.32 ± 12.11	65/106 (61.3%)		0	No nasal steroids, no antihistamine
Mu (2019)^22^	China	AR concomitant with asthma 65, HC 40	Not mentioned	AR 7.2 ± 1.9; HC7.2 ± 0.7	68/105 (64.8%)		100	Not mentioned
Wen (2019)^23^	China	PAR 90, HC 79	Not mentioned	AR: 9.7 ± 2.4; HC: 10.1 ± 1.9	116/169 (68.6%)		0	No corticosteroids, no leukotriene receptor antagonist
Liu (2020)^24^	China	AR 94, NAR 79, patients diagnosed with pituitary tumor or lateral skull base mass as HC 46	Symptomatic	AR: 30.5 ± 8.9; NAR: 37.7 ± 14.1; HC: 30.7 ± 5.5	160/219 (73.1%)		0	No corticosteroids

*Note*: Data is expressed in mean ± *SD* or mean (range).

Abbreviations: AR, allergic rhinitis; HC, healthy controls; n, number; NAR, nonallergic rhinitis; PAR, perennial allergic rhinitis; SAR, seasonal allergic rhinitis.

**Table 2:** Index test characteristics

Study, year	Analyzer	Flow rate (L/min)	Method	AR diagnosis
Henriksen (1999)^11^	LR200; Logan Research, Rochester, UK	0.25	BH	Reported AR and had a positive allergy screening blood test with sensitization to seasonal allergens
Palm (2003)^18^	Aerocrine AB, Stockholm, Sweden	0.5, 3, 9	ER	Ongoing, symptomatic and reportedly SPT positive no steroid‐treated birch pollen AR
Makris (2011)^19^	ECOmedics CLD88sp	3	ER	The diagnosis was based on the typical clinical symptoms and the documentation of sensitization with SPTs
Lee (2012)^7^	Sievers 280i (GE Analytical Instruments, Boulder, CO)	0.7	ER	Diagnosed with AR by history‐taking and multiple antigen simultaneous tests or skin tests
Suojalehto (2014)^20^	NIOX (Aerocrine AB, Solna, Sweden)	0.3	BH	At least one positive SPT and relevant rhinitis symptoms to that allergen
Nesic (2016)^8^	NIOX MINO (Aerocrine AB, Solna, Sweden)	0.3	BH	A history of more than 3 years of AR and were positive for serum allergen‐specific IgE against house dust mite or pollen
Hou (2018)^21^	NIOX MINO (Aerocrine AB, Solna, Sweden)	0.3	ER	At least 1 positive pollen IgE measurement and the presence of AR symptoms
Mu (2019)^22^	sunvou‐SU‐02E Analyzer	0.3	ER	Have the above clinical manifestations (symptoms, signs), and also have a positive result of any one of the 2 SPTs or serum specific IgE tests
Wen (2019)^23^	NIOX MINO (Aerocrine AB, Solna, Sweden)	0.3	BH	Defined according to the ARIA guidelines
Liu (2020)^24^	NIOX (Aerocrine AB, Sweden)	3	ER	Defined as a history of any of typical clinical symptoms with a positive SPT or serum specific IgE

Abbreviations: AR, allergic rhinitis; ARIA, allergic rhinitis and its impact on asthma; BH, breath hold; ER, exhalation against resistance; IgE, immunoglobulin E; SPT, skin prick test.

Different brands of NO analyzers were used in the included studies: NIOX, ECOmedics CLD88sp, LR200, NIOX MINO, Sievers 280i, Sunvou. Among them, NIOX MINO and Sunvou are electrochemical analyzers while the rest are chemiluminescence analyzers. During measurement of nNO, participants were required to obtain velum closure while gas was sampled from one nostril. Several methods including holding breath, exhaling against resistance can be achieved to ensure velum closure. Sampling flow rate is required to range from 0.25 to 3 L/min according to ATS recommendation.^12^

### 3.3 Quality assessment

**Figure 2:**
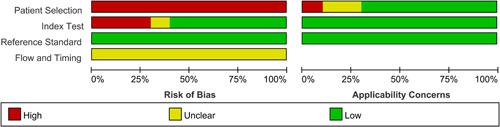
Methodological quality graph of each study with QUADAS‐2 tool for the 10 included studies. QUADAS‐2, Quality Assessment of Diagnostic Accuracy Studies‐2

**Figure 3:**
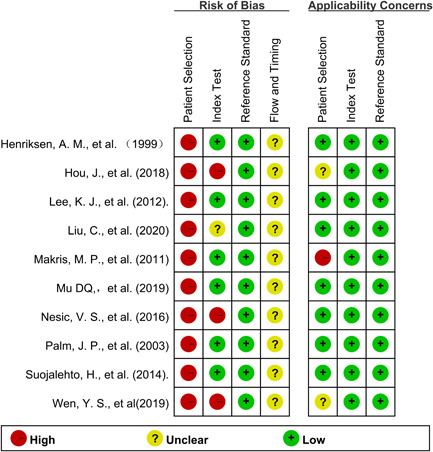
Methodological quality summary of each study with QUADAS‐2 tool for the 10 included studies. QUADAS‐2, Quality Assessment of Diagnostic Accuracy Studies‐2

The QUADAS‐2 tool was used to evaluate the internal and external validity of each study.^13^ The overall quality assessment was shown in the Figures 2 and 3. Because all studies recruited AR patients and healthy controls separately, the domain of patient selection had a high risk. Most studies did not mention the order of nNO measurement and AR diagnosis measurement. However, the domain of Index Test and Reference Standard had mostly low risk because those measurements were objective, a lack of blinding when evaluating these test results represented a smaller risk of bias. As for Flow and Timing domain, most studies did not mention the detailing time of nNO measurement and AR diagnosis measurement, so they mostly had unclear risk.

### 3.4 Ability of nNO to discriminate AR from healthy controls or NAR

**Figure 4:**
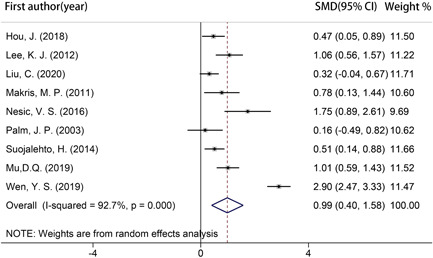
Forest plot showing the standardized mean differences in mean nasal nitric oxide between allergic rhinitis patients and healthy controls

Because only 1 study reported nNO values in media (range),^11^ 9 studies were included for meta‐analysis,^7^, ^8^, ^18^, ^19^, ^20^, ^21^, ^22^, ^23^, ^24^ which involved 515 AR patients, 315 healthy controls, and 123 NAR patients (Table 3). As shown in the Figure 4, AR patients represented significantly increased nNO compared with healthy controls (SMD: 0.989; 95% CI: 0.402, 1.576; *p* = .001). The heterogeneity of this outcome was significant (*I* ^2^ = 92.7%) and it did not decrease after individually eliminating each study. It could be relevant with analyzer types, sampling rates, sampling techniques or population characteristics.

**Table 3:** Summary of studies measuring nNO in AR, NAR, healthy controls groups following ATS standard

Study	AR		HC		NAR	
	Subjects *n*	Mean ± *SD* nl/min	Subjects *n*	Mean ± *SD* nl/min	Subjects *n*	Mean ± *SD* nl/min
Henriksen et al. (1999)^11^ ^,^ a	46	Median (range): 276.25 (137.75–512.75)	12	Median (range): 253.5 (122.5–408)		
Palm et al. (2003)^18^	18	160 ± 75 (flow: 0.5 L/min); 211 ± 103 (flow: 3 L/min)	18	153 ± 36 (flow: 0.5 L/min); 198 ± 45 (flow: 3 L/min)		
Makris et al. (2011)^19^	26	4162.4 ± 2895.9 (in pollen season); 3045.2 ± 1930.2 (out of pollen season)	15	2324.9 ± 538.7		
Lee et al. (2012).^7^	35	271.6 ± 83.4	34	193.5 ± 61.7		
Suojalehto et al. (2014).^20^	89	292.1 ± 82.8	42	253.3 ± 57.5	44	260.4 ± 86.5
Nesic et al. (2016)^8^	23	209.0 ± 40.8	10	138.2 ± 40.0		
Hou et al. (2018)^21^	75	61.7 ± 40.5	31	45.2 ± 14.5		
Mu et al. (2019)^22^	65	236.4 ± 126	40	133.2 ± 38.4		
Wen et al. (2019)^23^	90	229.6 ± 45.6	79	117.0 ± 29.2		
Liu et al. (2020)^24^	94	2817 ± 1005	46	2550 ± 309	79	2010 ± 564

Abbreviations: AR, allergic rhinitis; ATS, American Thoracic Society; HC, healthy controls; NAR, nonallergic rhinitis.

^a^ Studies not included in meta‐analysis due to the data not represented with mean ±* SD*.

Four studies^8^, ^21^, ^23^, ^24^ reported cut‐off values to discriminate between AR and healthy controls with their sensitivity and specificity (Table 4). Nesic et al.^8^ and Wen et al.^23^ used the same analyzer (NIOX MINO), the same flow rate (0.3 L/min) and the same method (BH), finally their nNO cut‐off value came out to be 169.4 and 161.4 nl/min, respectively. Their sensitivity was 83%, 100% and specificity was 80%, 94.9%, respectively.

**Table 4:** Studies presenting with cut‐off values for discriminating allergic rhinitis patients and healthy controls

Study	Analyzer	Flow rate (L/min)	Method	total subjects *n*	nNO cut‐off (nL/min)	sensitivity (%)	specificity (%)
Nesic et al. (2016)^8^	NIOX MINO	0.3	BH	AR 23, HC 10	169.4	83	80
Hou et al. (2018)^21^	NIOX MINO	0.3	ER	AR 75, HC 31	51.9	54.7	67.7
Wen et al. (2019)^23^	NIOX MINO	0.3	BH	AR 90, HC 79	161.4	100	94.9
Liu et al. (2020)^24^	NIOX	3	ER	AR 94, HC 46	2541	53.2	54.3

Abbreviations: AR, allergic rhinitis; BH, breath hold; ER, exhalation against resistance; HC, healthy controls; nNO, nasal nitric oxide.

**Figure 5:**
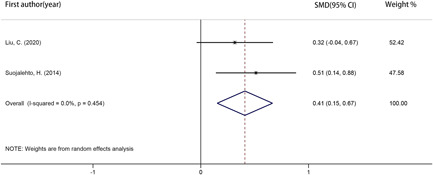
Forest plot showing the standardized mean differences in mean nasal nitric oxide between allergic rhinitis patients and nonallergic rhinitis patients

Two studies^20^, ^24^ reported nNO values of AR and NAR patients. As showed in the Figure 5, nNO value of AR patients was significantly higher than NAR (SMD: 0.680; 95% CI: 0.101, 1.259; *p* = .021). There was a high degree of heterogeneity (*I* ^2^ = 82.7%; *p* = .016).

### 3.5 Sensitivity analysis

After quality assessment of all studies, there were no studies of low risk bias to perform sensitivity analysis.

### 3.6 Subgroup analysis

Subgroup analysis was performed for studies measuring nNO with different measuring process and different patient characteristics. It was detailly shown in the Table 5.

**Table 5:** Subgroup analyses

	*N* of studies	*N* of participants	Effect size SMD (95% CI) in nNO
Studies of the different kinds of analyzers			
Stationary	5	262 AR; 155 HC	SMD: 0.554; 95% CI: 0.260, 0.849; *p* = .010; *I* ^2^ = 46.3%, *p* = .114
Handheld	4	253 AR; 160 HC	SMD: 1.526; 95% CI: 0.361, 2.691; *p* = .010; *I* ^2^ = 95.6%, *p* = .000
Studies of the different sampling techniques			
BH	3	202 AR; 131 HC	SMD: 1.717; 95% CI: 0.029, 3.404; *p* = .046; *I* ^2^ = 97.0%, *p* = .000
ER	6	313 AR; 184 HC	SMD: 0.638; 95% CI: 0.337, 0.938; *p* = .000; *I* ^2^ = 57.3%, *p* = .039
Studies of the different sampling flow rates			
3 L/min	3	138 AR; 79 HC	SMD: 0.374; 95% CI: 0.092, 0.656; *p* = .009; *I* ^2^ = 0.0%, *p* = .369
0.3 L/min	5	342 AR; 202 HC	SMD: 1.316; 95% CI: 0.368, 2.264; *p* = .007; *I* ^2^ = 95.3%, *p* = .000
Studies of the AR patients with/without asthma			
With asthma	1	65 AR; 40 HC	SMD: 1.011; 95% CI: 0.593, 1.428; *p* = .000; *I* ^2^ not applicable, *p* not applicable
Without asthma	8	450 AR; 275 HC	SMD: 0.987; 95% CI: 0.310, 1.665; *p* = .004; *I* ^2^ = 93.6%, *p* = .000
Studies of the AR patients with/without rhinitis symptoms			
Having rhinitis symptoms	4	213 AR; 110 HC	SMD: 0.404; 95% CI: 0.169, 0.638; *p* = .001; *I* ^2^ = 0.0%, *p* = .545
Not sure having rhinitis symptoms	5	302 AR; 205 HC	SMD: 1.438; 95% CI: 0.529, 2.346; *p* = .002; *I* ^2^ = 94.5%, *p* = .000
Studies of the AR patients with/without nasal polyps			
With nasal polyps	1	10 AR; 42 HC	SMD: −0.215; 95% CI: −0.905, 0.476; *p* = .543; *I* ^2^ not applicable, *p* not applicable
Without nasal polyps	5	361 AR; 208 HC	SMD: 1.195; 95% CI: 0.200, 2.189; *p* = .019; *I* ^2^ = 96%, *p* = .000
Studies of the AR patients with/without sinusitis			
With sinusitis	2	77 AR; 125 HC	SMD: 0.972; 95% CI: −3.627, 5.571; *p* = .679; *I* ^2^ = 99.3%, *p* = .000
Without sinusitis	5	246 AR; 161 HC	SMD: 1.102; 95% CI: 0.689, 1.515; *p* = .000; *I* ^2^ = 70.5%, *p* = .009
Studies of AR patients excluding/not excluding smoking			
Excluding smoking	4	142 AR; 74 HC	SMD: 0.723; 95% CI: 0.174, 1.272; *p* = .010; *I* ^2^ = 67.2%, *p* = .027
Not excluding smoking	5	373 AR; 241 HC	SMD: 1.157; 95% CI: 0.264, 2.049; *p* = .011; *I* ^2^ = 95.8%, *p* = .000
Studies of AR patients with/without marked ostial obstruction			
With marked ostial obstruction	2	41 AR; 73 HC	SMD: −0.668; 95% CI: −1.498, 0.161; *p* = .114; *I* ^2^ = 72.5%, *p* = .057
Without marked ostial obstruction	2	123 AR; 73 HC	SMD: 0.950; 95% CI: 0.252, 1.647; *p* = .2016; *I* ^2^ = 79.3%, *p* = .028

Abbreviations: AR, allergic rhinitis; BH, breath hold; CI, confidence interval; ER, exhalation against resistance; HC, healthy controls; N, number; nNO, nasal nitric oxide; SMD, standardized mean differences.

Comparison 1: subgroup analysis by different NO analyzer types

In the subgroup analysis, we analyzed the effects of NO analyzer types on nNO's ability to discriminate AR. There was no evidence for different effects of NO analyzer types between subgroups.

Comparison 2: subgroup analysis by different NO sampling techniques

In the subgroup analysis, we analyzed the effects of NO sampling techniques on nNO's ability to discriminate AR. There was no evidence for different effects of NO sampling techniques between subgroups.

Comparison 3: subgroup analysis by different NO sampling flow rates

In the subgroup analysis, we analyzed the effects of NO sampling flow rates on nNO's ability to discriminate AR. There was no evidence for different effects of NO sampling flow rates between subgroups.

Comparison 4: subgroup analysis by AR patients with/without asthma

In the subgroup analysis, we analyzed the concomitant of asthma on nNO's ability to discriminate AR. There was no evidence for different effects of asthma between subgroups.

Comparison 5: subgroup analysis by AR patients with/without rhinitis symptoms

In the subgroup analysis, we analyzed the existence of rhinitis symptoms on nNO's ability to discriminate AR. There was no evidence for different effects of rhinitis symptoms between subgroups.

Comparison 6: subgroup analysis by AR patients with/without nasal polyps

In the subgroup analysis, we analyzed the existence of nasal polyps on nNO's ability to discriminate AR. There was evidence for different effects of nasal polyps between subgroups. No significant differences of nNO were detected between AR patients with nasal polyps and healthy controls.

Comparison 7: subgroup analysis by AR patients with/without sinusitis

In the subgroup analysis, we analyzed concomitant of sinusitis on nNO's ability to discriminate AR. To be more specific, sinusitis meant acute unilateral maxillary sinusitis or sinus inflammation in the studies enrolling AR patients with sinusitis.^23^, ^24^ There was evidence for different effects of sinusitis between subgroups. No significant differences of nNO were detected between AR patients with sinusitis and healthy controls.

Comparison 8: subgroup analysis by AR patients excluding/not excluding smoking

In the subgroup analysis, we analyzed smoking on nNO's ability to discriminate AR. There was no evidence for different effects of smoking between subgroups.

Comparison 9: subgroup analysis by AR patients with/without marked ostial obstruction

In the subgroup analysis, we analyzed the existence of marked ostial obstruction on nNO's ability to discriminate AR. The ostial obstruction was measured through a semiquantitative computed tomography scoring system or active anterior rhinomanometry.^20^, ^21^There was evidence for different effects of marked ostial obstruction between subgroups. No significant differences of nNO were detected between AR patients with marked ostial obstruction and healthy controls.

### 3.7 Publication bias

**Figure 6:**
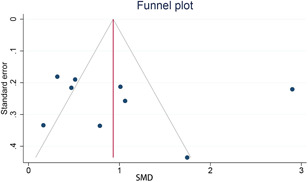
Funnel plot for studies evaluating nasal nitric oxide in allergic rhinitis patients and healthy controls

Publication bias was detected by visual examination to funnel plot (Figure 6). While the Egger test (*p* = .7251) and the Begg and Mazumdar test (*p* = .1179) indicated no publication bias.

## 4 DISCUSSION

In this systematic review and meta‐analysis, we have shown that nNO in AR patients was significantly higher than healthy controls and NAR. The nNO measurement in accordance with ATS recommended standardized procedure fits for kids older than 4 years old and adults who are able to cooperate with sampling techniques to ensure velum closure. Subgroup analysis showed that when AR patients were concomitant with nasal polyps, sinusitis or marked ostial obstruction, it was hard for nNO to detect them.

Gustafsson's group found endogenous NO was present in the exhaled air of humans and other mammals in 1991.^25^ NO is synthesized from l‐arginine by NO synthase (NOS) in the respiratory system, which has three isoforms: neuronal NOS (nNOS), inducible NOS (iNOS), and endothelial NOS (eNOS).^26^ iNOS is induced by proinflammatory cytokines and/or bacterial products in almost every epithelial cell, while the other two isoforms are constitutively expressed.^6^, ^26^, ^27^ So the level of exhaled NO is usually increased and regulated by iNOS enzyme.^28^ Studies discovered that NO in the exhaled air of patients with asthma was higher than healthy controls.^29^, ^30^ Now, serving as an indicator of eosinophil inflammation of the lower respiratory tract, high level of FeNO suggests possibility of asthma in National Institute for Health and Care Excellence (NICE): Clinical Guidelines.^3^ Furthermore, FeNO has been used to guide inhaled corticosteroid treatment in asthma patients and chronic cough patients.^5^, ^31^, ^32^ Compared with FeNO value in lower airway, nNO has a far higher level in upper airway, which had been proofed by Alving in 1993.^33^ Most studies indicated that the main production of nNO was in paranasal sinuses. As the epithelium of sinuses produces a large amount of NO,^34^, ^35^, ^36^ it may explain the reason why nNO value is far higher than FeNO.

There are two different ways of measuring the fractional concentration of nasal NO. If the measurement is obtained by nasal exhalation, it is called nasal FeNO. If the measurement is obtained by transnasal flow in series, it is called nNO.^6^ Our study only focused on nNO because it is recommended by ATS.^12^ In this meta‐analysis, we found that nNO in AR was significantly higher than healthy controls or NAR. It was consistent with the finding in eosinophilic chronic rhinosinusitis that higher levels of nasal FeNO may reflect the persistence of eosinophilic inflammation in sinus mucosa with concomitant iNOS upregulation.^37^ However, some studies reported nNO was not statistically different in AR compared with healthy controls. Swelling of nasal mucosa may lead to occluded sinus ostia and then prevent NO distributing to nasal cavity, which may explain the contradicting results. Wen et al.^23^ found that nNO level in PAR patients with acute maxillary sinusitis was negatively correlated to total nasal resistance. Hou et al.^21^ found that nNO in AR patients with nasal obstruction score more than 7 was significantly decreased compared with healthy subjects, while nNO in AR patients with nasal obstruction score less than 7 was significantly increased compared with healthy subjects.^21^ These studies and our finding may explain the reason of current controversial study results. We did subgroup analysis for different patient characteristics and measuring process. Subgroup analysis in different patient characteristics showed that nNO could not detect AR patients concomitant with nasal polys, sinusitis or marked ostial obstruction. The rest factors, including different analyzer types, sampling flow rates, sampling techniques, concomitant asthma, rhinitis symptoms and smoking, do not impair nNO's ability in discriminating AR from healthy controls.

Using the same analyzer, same flow rate and same method, Nesic et al.^8^ and Wen et al.^23^ reported similar cut‐off value (169.4 and 161.4 nl/min, respectively) with good specificity and sensitivity, which means experts could set a specific cut‐off value under single specific nNO measuring procedure for AR screening.

Our study presented with some limitations. First, high degree heterogeneity significantly influences our results. Although it is hard to determine the exact source of heterogeneity, here are some possible sources: included studies were held in different countries and different inclusion and exclusion criteria were set, leading to diverse demographic and clinical characteristics; few studies gave detailed description on AR patients such as their AR symptoms; kids were involved in meta‐analysis, while nNO were age‐related in kids younger than 12 years old.^38^ Second, all included studies were case‐control designed, studies reporting cut‐off values did not prespecify threshold, both causing it potentially overestimate the accuracy of a diagnostic test.

All considered, our meta‐analysis found that nNO in AR patients are significantly higher than healthy controls and NAR. nNO serves as a potential indicator for discriminating AR. However, nasal polyps, sinusitis and marked ostial obstruction are supposed to be taken into consideration before nNO is applied to detect AR. In addition, referring to the role of FeNO played in asthma, it remains to be seen whether nNO could be used as an indicator of AR treatment responsiveness in future studies.

## CONFLICT OF INTERESTS

The authors declare that there are no conflict of interests.

## AUTHOR CONTRIBUTIONS

All author drafted the manuscript. Conception was from Bingbing Wang, Zhenchao Wu, and Yi Liu. Bingbing Wang designed the study protocol, performed data extraction and checked the manuscript. Zhenchao Wu performed data extraction, data analysis and interpretation, revised the manuscript to make it more fluent. Feifei Wang, Zuojuan Yin and Lei Shi did the literature search and study selection. Administrative support from Yi Liu who was responsible for any disagreement arising from the whole work.

## ETHICS STATEMENT

I confirm that the manuscript has been submitted solely to this journal and is not published, in press, or currently submitted elsewhere.

## Supporting information

Supporting information.

## Data Availability

The data that support the findings of this study are available from the corresponding author upon reasonable request.
